# Tumour marker antigens during menses and pregnancy.

**DOI:** 10.1038/bjc.1989.297

**Published:** 1989-09

**Authors:** Y. Touitou, Y. Darbois, A. Bogdan, A. AuzÃ©by, S. Keusseoglou

**Affiliations:** Department of Biochemistry, FacultÃ© de MÃ©decine PitiÃ©-SalpÃªtriÃ¨re, Paris, France.


Br. J. Cancer (1989), 60, 419–420 © The Macmillan Press Ltd., 1989

SHORT COMMUNICATION

### Tumour marker antigens during menses and pregnancy

Y. Touitou, Y. Darbois, A. Bogdan, A. Auzéby & S. Keusseoglou

*Department of Biochemistry, Faculté de Médecine Pitié-Salpêtrière, 91 boulevard de l'Hôpital, 75634 Paris Cedex 13, France.*

The specificity of tumour markers in cancer is poor. Indeed, a number of pathophysiological occurrences can induce significant variations of tumour marker plasma concentrations (Touitou & Bogdan, 1988*a*, *b*). The situations resulting in false positive determination have thus to be kept in mind to avoid any misinterpretation in the follow-up of the patients. It has been recently reported that in women, CA 125 plasma concentrations increased during pregnancy (Niloff *et al*., 1984) and menstruation (Haga *et al*., 1986; Jäger *et al*., 1988). Although infrequent, pregnancy does occur in women with a cancer. Therefore, we have documented the serum concentrations of a set of routinely determined tumour markers (CEA, CA-125, CA-19.9, CA-15.3 and SCC) in healthy pregnant women taking into account the stage of pregnancy and in young women at two stages of their menstrual cycle.

A transverse study was carried out on 100 unselected pregnant women. Blood samples were obtained at the time of routine examinations during pregnancy. The subjects were grouped according to the stage of pregnancy (weeks of amenorrhea), i.e. 8.5–13 weeks: 32 women (mean age: $29.2 \pm 0.9$); 13.5–26 weeks: 32 women (mean age: $27.8 \pm 1.1$) and 26.5–42 weeks: 36 women (mean age: $30.6 \pm 0.6$). A parallel study was carried out on 22 young women volunteers (medical students, mean age: $21.8 \pm 1.4$) with blood samplings at day 2 and day 9 of their menstrual cycle.

Venous blood samples (without anticoagulant) were drawn under standardised conditions without haemolysis, from the antecubital vein and the serum centrifuged. The serum aliquots were kept at $-20$ °C until assayed. Commercial kits were used for the determination of the following tumour markers: SCC (RIA, Abbott), CEA (EIA, Abbott), CA-125, CA-19.9 and CA-15.3 (IRMA, CIS). The assays were performed in the same series to avoid inter-assay variations. The intra-assay coefficient of variations ($n = 30$) were as follows: CEA, 7.1% at 3 ng ml$^{-1}$; CA-125, 8.7% at 45 U ml$^{-1}$; CA-19.9, 7.1% at 25 U ml$^{-1}$; CA-15.3, 3.5% at 25 U ml$^{-1}$; SCC, 11.3% at 3 ng ml$^{-1}$. Statistical analysis was performed using Student's *t* test (two-tailed).

In the 22 young control women the plasma concentrations of tumour markers at day 2 were all within the normal range except for CA-19.9, which was found abnormally high in three cases (range 43–65 U ml$^{-1}$). No changes were observed at day 9 when compared to day 2 (Tables I and II).

In pregnant women the plasma concentrations of the markers were, as a rule, found within the normal range except two slightly elevated SCC (2.3 and 2.5 ng ml$^{-1}$) and especially ten CA-19.9 (38–117 U ml$^{-1}$), i.e. for this latter: three cases (9.4%) ranging from 38 to 434 U ml$^{-1}$ in the first trimester, five cases (15.6%) ranging from 47 to 67 U ml$^{-1}$ in the second trimester and two cases (5.6%) with values of 71 and 117 U ml$^{-1}$ in the third trimester of pregnancy. CA-15.3 plasma concentration was found to increase significantly (from $11.1 \pm 4.2$ to $17.0 \pm 5.0$; mean ± s.d.) with the stage of pregnancy (Tables I and II). SCC plasma concentration was also significantly higher in the second and third trimesters (1.25 and 1.10 ng ml$^{-1}$ respectively) when compared to the first trimester of pregnancy (0.77 ng ml$^{-1}$). The CA-125 determination showed four cases (12.5%) with elevated concentrations in the first trimester of pregnancy, one (3.1%) in the second trimester and three (8.3%) in the third trimester.

All the tumour markers documented in this report are of common use in monitoring cancers, namely breast (CA-15.3 and CEA), ovarian (CA-125), pancreatic (CA-19.9), colon and rectum cancers (CEA and CA-19.9) and squamous cell carcinomas, e.g. uterine cervix (SCC). We report here the effect of menses on the mean plasma concentrations of CA-125 in 10 healthy young women, i.e. a rise at day 2 (15.4 U ml$^{-1}$) when compared with day 9 (9.7 U ml$^{-1}$). We could thus confirm the results from Pittaway & Fayez (1987), who reported in nine women an increase from $13 \pm 3$ U ml$^{-1}$ (days 23–26 of cycle) to $29 \pm 7$ U ml$^{-1}$ (days 2–4 of cycle) of CA-125 plasma concentrations. However, neither in the women studied by these authors nor in those studied here did this increase reach the cut-off level of 35 U ml$^{-1}$. All the other documented markers were not found modified during menses.

This transverse study on pregnant women allowed us to show that CA-15.3 and SCC plasma concentrations increased significantly during pregnancy although remaining within the normal range. No differences could be seen between the three stages of pregnancy for plasma mean concentrations of the other documented tumour markers (CEA and CA-19.9).

Nevertheless it has to be emphasised that 10% of the studied pregnant women had abnormally high CA-19.9 plasma concentrations (38–117 U ml$^{-1}$), without any apparent relation to the stage of pregnancy. In the same way, three control women (13.6%), in apparent good health, had abnormally high plasma concentrations of this marker (43–65 U ml$^{-1}$). CA-19.9 is a poorly specific marker since plasma concentrations have been reported to be increased in a number of non-malignant diseases (Touitou & Bogdan, 1988*a*). In the present study we could not attribute the observed increases to any detectable benign pathology. The determination of plasma concentrations of CA-125 in pregnant women confirmed the rise of plasma CA-125 reported in the literature: Nilof *et al*. (1984) found elevated levels of this marker in six out of 38 women (16%) in the first trimester of pregnancy, whereas Halila *et al*. (1986) and Pittaway & Fayez (1987) found respectively 11 out of 46 (24%) and three out of 15 women (20%) with increased CA-125 plasma levels, disregarding the stage of pregnancy.

In conclusion, CA-125, CA-15.3 and SCC appear to deserve special attention when documented in pregnant women, and CA-19.9 seems to be very sensitive to benign diseases and care should be given to the interpretation of results of this tumour marker.

This work was supported by La Caisse Régionale d'Assurance Maladies d'Ile de France and le Conseil Scientifique de l'Université P. & M. Curie (Formation Recommandée par la Direction de la Recherche). We wish to thank Abbot Laboratories (Rungis, France) and CIS-International (Gif-sur-Yvette, France) for their help in this study.

Correspondence: Y. Touitou.
Received 27 February 1989, and in revised form, 3 May 1989.

**Table I** Tumour markers in pregnancy and in non-pregnant women at two stages of their menstrual cycle

| | n | *Age (years)* ± *s.d.* | *CEA* ($ng\,ml^{-1}$) ± *s.d.* | *CA 15.3* ($U\,ml^{-1}$) ± *s.d.* | *CA 125* ($U\,ml^{-1}$) ± *s.d.* | *SCC* ($ng\,ml^{-1}$) ± *s.d.* | *CA 19.9* ($U\,ml^{-1}$) ± *s.d.* |
|---|---|---|---|---|---|---|---|
| Pregnancy in weeks of amenorrhea | | | | | | | |
| ≤13 | 32 | 29.2±4.9 | 0.65±0.35 | 11.1±4.2 [b] | 23.7±13.9 [b] [c] | 0.77±0.60 [c] | 17.8±11.0 |
| 13.5–26 | 32 | 27.8±6.4 | 0.59±0.21 | 14.2±5.1 [a] | 14.8±8.0 [b] | 1.25±0.37 | 18.8±17.3 |
| 26.5–42 | 36 | 30.6±3.4 | 0.57±0.23 | 17.0±5.0 | 22.1±17.1 | 1.10±0.56 | 16.7±20.8 |
| Control women during menstrual cycle | | | | | | | |
| Day 2 | 22 | 21.8±6.1 | 1.11±0.71 | 12.9±4.1 | 15.4±7.6[d] | 1.80±0.44 | 22.7±16.9 |
| Day 9 | 22 | | 1.04±0.66 | 12.2±3.4 | 9.7±5.0[d] | 1.71±0.39 | 22.9±14.5 |

Means are different (two-tailed *t* test) with: [a]$P<0.05$; [b]$P<0.01$; [c]$P<0.001$; [d]Ten subjects only.

**Table II** Percentage of false positive rate of tumour marker antigens in pregnancy and during menses

| | *Number (and percentage) of subjects above the cut-off value* | | | | | |
|---|---|---|---|---|---|---|
| | *n* | *CEA* | *CA-15.3* | *CA-125* | *SCC* | *CA-19.9* |
| Pregnancy in weeks of amenorrhea | | | | | | |
| All | 100 | 0 | 0 | 8(8.0) | 2(2.0) | 10(10.0) |
| ≤13 | 32 | 0 | 0 | 4(12.5) | 0 | 3(9.4) |
| 13.5–26 | 32 | 0 | 0 | 1(3.1) | 1(3.1) | 5(15.6) |
| 26.5–42 | 36 | 0 | 0 | 3(8.3) | 1(2.8) | 2(5.6) |
| Control women | 22 | 0 | 0 | 1(10.0)[a] | 0 | 3(13.6) |

[a]Ten subjects only.

### References

HAGA, Y., SAKAMOTO, K. HIROSHI, E., YOSHIMURA, R. & AKAGI, M. (1986). Evaluation of serum CA-125 values in healthy individuals and pregnant women. *Am. J. Med. Sci.*, **292,** 25.

HALILA, H., STENMAN, U.H. & SEPPÄLÄ, M. (1986). Ovarian cancer antigen CA 125 levels in pelvic inflammatory diseases and pregnancy. *Cancer*, **57,** 1327.

JÄGER, W., MEIER, C., WILDT, L., SAUERBREI, W. & LANG, N. (1988). CA 125 concentrations during the menstrual cycle. *Fertil. Steril.*, **50,** 223.

NILOFF, J.M., KLUG, T.L., SCHAETZL, E., REYNOLDS, C. & BAST, R.C. (1984). CA 125 antigen in obstetric and gynecologic patients. *Obstet. Gynecol.*, **64,** 703.

PITTAWAY, D.E. & FAYEZ, J.A. (1987). Serum CA 125 levels increase during menses. *Am. J. Obstet. Gynecol.*, **156,** 75.

TOUITOU, Y. & BOGDAN, A. (1988*a*). Tumor markers in non-malignant pathologies. *Eur. J. Cancer Clin. Oncol.*, **24,** 1083.

TOUITOU, Y. & BOGDAN, A. (1988*b*). Etude critique des marqueurs tumoraux récents. *Bull. Cancer*. **75,** 247.


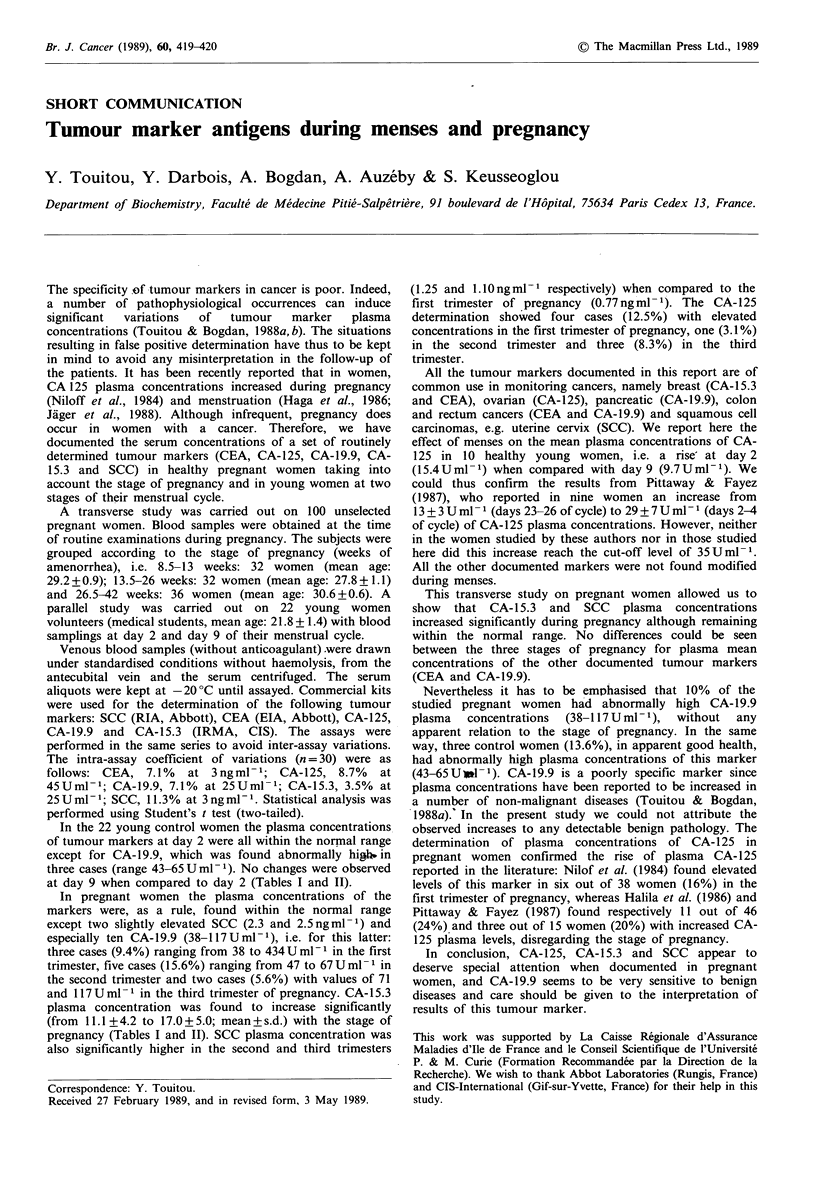

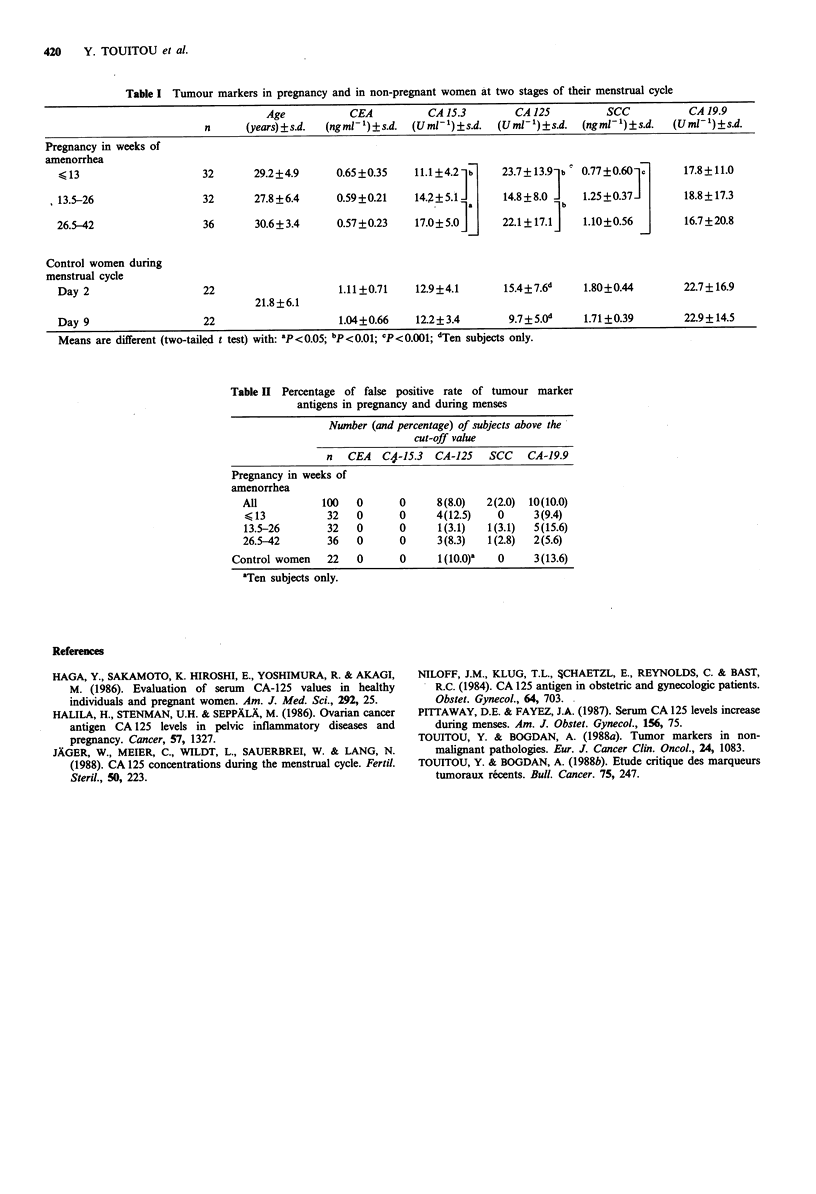


## References

[OCR_00199] Halila H., Stenman U. H., Seppälä M. (1986). Ovarian cancer antigen CA 125 levels in pelvic inflammatory disease and pregnancy.. Cancer.

[OCR_00204] Jäger W., Meier C., Wildt L., Sauerbrei W., Lang N. (1988). CA-125 serum concentrations during the menstrual cycle.. Fertil Steril.

[OCR_00209] Niloff J. M., Knapp R. C., Schaetzl E., Reynolds C., Bast R. C. (1984). CA125 antigen levels in obstetric and gynecologic patients.. Obstet Gynecol.

[OCR_00214] Pittaway D. E., Fayez J. A. (1987). Serum CA-125 antigen levels increase during menses.. Am J Obstet Gynecol.

[OCR_00222] Touitou Y., Bogdan A. (1988). Etude critique des marqueurs tumoraux récents.. Bull Cancer.

[OCR_00218] Touitou Y., Bogdan A. (1988). Tumor markers in non-malignant diseases.. Eur J Cancer Clin Oncol.

